# Finding My Way: protocol of a randomised controlled trial evaluating an internet self-help program for cancer-related distress

**DOI:** 10.1186/s12885-015-1322-x

**Published:** 2015-04-30

**Authors:** Lisa Beatty, Emma Kemp, Tracey Wade, Bogda Koczwara, on behalf of the Finding My Way study investigators

**Affiliations:** 1 School of Psychology, Flinders University, GPO Box 2100, Adelaide, SA 5001 Australia; 2 Flinders Centre for Innovation in Cancer, Flinders Medical Centre, Flinders Drive, Bedford Park, SA 5042 Australia; 3 School of Medicine, Flinders University, GPO Box 2100, Adelaide, SA 5001 Australia

**Keywords:** Cancer, Psycho-oncology, Internet, Intervention, Distress, Coping, CBT, RCT

## Abstract

**Background:**

A cancer diagnosis elicits greater distress than any other medical diagnosis, and yet very few studies have evaluated the efficacy of structured online self-help therapeutic programs to alleviate this distress. This study aims to assess the efficacy over time of an internet Cognitive Behaviour Therapy (iCBT) intervention (‘*Finding My Way*’) in improving distress, coping and quality of life for individuals with a recent diagnosis of early stage cancer of any type.

**Methods/Design:**

The study is a multi-site Randomised Controlled Trial (RCT) seeking to enrol 188 participants who will be randomised to either the *Finding My Way* Intervention or an attention-control condition. Both conditions are delivered online; with 6 modules released once per week, and an additional booster module released one month after program-completion. Participants complete online questionnaires on 4 occasions: at baseline (immediately prior to accessing the modules); post-treatment (immediately after program-completion); then three and six months later. Primary outcomes are general distress and cancer-specific distress, with secondary outcomes including Health-Related Quality of Life (HRQoL), coping, health service utilisation, intervention adherence, and user satisfaction. A range of baseline measures will be assessed as potential moderators of outcomes. Eligible participants are individuals recently diagnosed with any type of cancer, being treated with curative intent, aged over 18 years with sufficient English language literacy, internet access and an active email account and phone number. Participants are blinded to treatment group allocation. Randomisation is computer generated and stratified by gender.

**Discussion:**

Compared to the few prior published studies, *Finding My Way* will be the first adequately powered trial to offer an iCBT intervention to curatively treated patients of heterogeneous cancer types in the immediate post-diagnosis/treatment period. If found efficacious, *Finding My Way* will assist with overcoming common barriers to face-to-face therapy in a cost-effective and accessible way, thus helping to reduce distress after cancer diagnosis and consequently decrease the cancer burden for individuals and the health system.

**Trial registration:**

Australian New Zealand Clinical Trials Registry ACTRN12613000001796 16.10.13

## Background

Despite improvements in treatment and survival rates, a cancer diagnosis continues to elicit greater distress than any other medical diagnosis [1]. Research has consistently found that 30 to 40 per cent of recently diagnosed patients experience clinically significant depressive or anxiety disorders [2,3]. Distress is most acute during the first 12 months after diagnosis, when a range of medical treatments take place, following which the prevalence reduces to levels comparable with the community [4]. Consequently, distress management within the first 12 months after diagnosis has been recognised as an integral component of a patient’s clinical treatment [5,6]. Despite this, distress and psychiatric disorders among cancer patients often go under-recognised and untreated [7]. This particularly applies to the 30% of Australian cancer patients who reside in rural or regional areas, where access to specialist cancer services is limited [6].

### Psychological approaches that alleviate distress

Over the past two decades, a range of psychological interventions to alleviate distress have been empirically investigated, predominantly among women with breast cancer. Intervention research has focused on developing and evaluating interventions that target the known psychological predictors of maladjustment [8], namely, low social support, prior anxiety and depression (vulnerability to distress), and maladaptive coping, particularly repression of emotions, helplessness/hopelessness, and cognitive and behavioural avoidance. Interventions tested have included those based on Cognitive Behavioural Therapy (CBT) [9], Supportive Expressive Therapy (SET) [10], psycho-educational programs [11], mindfulness-based therapy [12], and peer-support groups [13]. There is clear meta-analytic evidence that all of these conventional psychosocial interventions are efficacious compared to control groups in facilitating adjustment [14]; with large effect sizes (ES) found for depression (ES = 1.2), anxiety (ES = 1.99), and functional adjustment / quality of life (QOL) (ES = 0.91) [14]. However, CBT has the largest evidence base for treating cancer-related distress to date [14], with most studies suggesting that treatment for distress at the time of cancer diagnosis is more effective than after cancer treatment has been completed [11].

### Need for novel approaches to improve access

While demonstrably efficacious, there several barriers to attending face-to-face therapy in the Australian setting. First, there are limited psychosocial services freely available in the public health setting, with the current capacity not sufficient to meet the demand [6]. This is particularly evident in rural and remote areas where access to multidisciplinary cancer care is problematic, and resulting inequities in health outcomes are well recognised [6]. Second, even when psychosocial services are offered in urban settings, research suggests that less than 25% of Australians attend [15], with low attendance attributed to a variety of barriers including: personal barriers, such as ongoing stigma associated with seeking mental health assistance [16,17], and illness-related barriers such as patients’ reduced physical capacity for additional appointments [17]. As a result of these barriers, national government supportive care policies have recognised that innovative methods of increasing access to supportive care throughout the treatment trajectory are required for all cancer patients [6].

One such innovative method of increasing patient access to supportive care is through internet-based provision of psychosocial services [16-18]. Since 2000 a plethora of randomised controlled trials and systematic reviews have examined internet-based interventions for both psychological [19,20] and physical [17,18] health complaints. Of these interventions, self-guided web-based interventions offer the particular advantage of overcoming the majority of the barriers discussed above for both rural and urban patients, along with anonymity, privacy and convenience of progressing through a program at the user’s own pace. Such interventions tend to be highly structured into modules, are derived from theory (predominantly cognitive behaviour therapy), and have been adapted from existing evidence-based print-resources or face-to-face therapy [21]. Consistent with research on face-to-face therapy, internet Cognitive Behaviour Therapy (iCBT) interventions have the largest evidence base for efficacy in improving both mental health conditions [22] and, increasingly, coping with chronic physical health conditions [17,18].

### Status of iCBT for cancer distress

Given that cancer consumers prefer multi-media to print resources [23], and that 84% of Australians have access to the internet [24], web-based interventions, and in particular iCBT-based interventions, appear a logical format for the wide dissemination of psychological treatment for cancer patients. However, the evidence-base for self-guided iCBT-based psychological therapy in cancer is only now emerging [25-28].

Two groups [26,28] developed and evaluated structured self-guided online CBT-based coping programs for women with recently diagnosed early-stage breast cancer. Similar in design, their programs were both multi-component, and contained 6–10 self-guided CBT-based modules, biomedical information, links to other cancer resources and websites, and asynchronous discussion boards. Both studies showed evidence for the efficacy of their respective interventions: The smaller of the two studies [28] found one significant interaction where women with poorer baseline perceived health status experienced greater improvements in perceived health over time when assigned to the intervention, despite a small sample and consequent lack of power. The larger study [26] found the intervention was efficacious in improving self-efficacy for coping with cancer, regulating negative mood, and reducing cancer-related post-traumatic symptoms. However, as each of these studies involved only breast cancer patients, efficacy could not be demonstrated across cancer types.

In the third study, Duffecy et al. [27] ran a small RCT of 31 post-treatment survivors of various cancer types to examine the feasibility and acceptability of an 8-week Internet Support Group (ISG) combined with an Individual Internet Intervention (III), compared with the III alone. The study supported feasibility and acceptability of the combined program, and found that both conditions produced large reductions in depressive symptoms in participants with high baseline distress. However, the study was underpowered to detect improvement over time and time x treatment effects; further, it examined efficacy in post-treatment survivors, rather than patients currently undergoing treatment. Current CBT literature suggests that a more effective time to implement such interventions may be in the immediate post-diagnosis period, when patients are likely to be experiencing higher levels of distress generally [4].

Our research group was the first to pilot test a 6-week iCBT program for people with any type of early stage/curatively treated cancer, and designed to be delivered soon after diagnosis [25]. In contrast to the previous three programs which all depended on group-support as a key feature, our study tested an individual self-guided web-based intervention for newly diagnosed patients that was independent of moderators and peer-support components, and was therefore consistent with the large evidence base for CBT [14]. Program content was derived from our evidence-based print self-help breast cancer workbook [29], but heavily revised and expanded to ensure relevance to heterogeneous cancer populations. In a case-series design, participants (n = 12) were assessed at baseline and after completing the program. Analysis found the intervention led to reductions with moderate associated effect sizes in negative affect, helplessness/hopelessness, anxious preoccupation, and fatalism. Results therefore provided preliminary support for the potential efficacy of the web-based CBT program for improving cancer-related distress and anxious preoccupation after cancer diagnosis. However, the pilot nature of this study and small sample size meant that the efficacy of the intervention compared with a control condition could not be established.

In summary, the four studies published to date provide preliminary evidence of the feasibility of iCBT interventions for delivering psychosocial interventions for coping with cancer. However, studies examining the efficacy of iCBT programs aimed at heterogeneous cancer types have so far been limited by either (1) small sample sizes and consequent lack of power, (2) lack of a control condition, and/or (3) not providing analysis of the longer-term impact of the intervention. Further, only our group have investigated a self-guided intervention that is (a) independent of discussion board components and therefore truly self-guided and (b) applicable to delivery soon after diagnosis, as opposed to post cancer treatment, and therefore consistent with CBT literature suggesting that treatment for distress at the time of cancer diagnosis is more effective than treatment for distress after treatment for cancer.

This paper reports on the protocol for the proposed RCT to examine the effectiveness of the iCBT intervention in a multisite setting.

### Aims and hypotheses

This study aims to evaluate the efficacy of the next iteration of our self-help internet cancer coping intervention (‘*Finding My Way’*) in improving wellbeing and quality of life of early-stage cancer patients over time, using a multi-site RCT.

A secondary aim is to investigate moderators of therapeutic change, consistent with recommendations that RCTs should identify who benefits most, and under what conditions, from psychosocial interventions [30]. For the present study, factors that have been previously shown to predict distress will be examined for their potential to moderate the effects of the intervention; namely, availability of social support, cognitive vulnerability to distress, and motivation to seek information [8,31].

Therefore it is hypothesised that:

1. Intervention participants will report greater improvements compared to attention-control participants in the primary outcome, distress, from baseline to post-treatment and 3- and 6-month follow-up;
2. Intervention participants will report greater improvements compared to attention-control participants in the secondary outcomes coping and HRQOL over time;
3. Primary and secondary outcomes will be moderated such that
  - The significant changes over time in distress between groups will be moderated by social support, cognitive vulnerability to distress, and motivation to seek information, and
  - The significant changes over time in coping and HRQOL between groups will be moderated by social support, cognitive vulnerability to distress, and motivation to seek information.

## Methods/Design

### Study design

Finding My Way (FMW) is a national level multi-site randomised controlled trial registered with the Australian and New Zealand Clinical Trials Registry with the registration number ACTRN12613000001796. Ethical approval to conduct the study has been sought and obtained from the Southern Adelaide Clinical, Royal Brisbane and Women’s and ACT Health Human Research Ethics Committees. Randomisation occurs at the patient level: patients are randomised to receive either the FMW intervention or an online attention-control. Procedural elements of this study follow CONSORT guidelines [32]. Attrition is monitored and reasons for non-participation and withdrawal are recorded in each arm. Data extraction and entry will be performed by research team members blinded to group allocation.

### Setting

Participation in the study occurs entirely online. The web-program is hosted by Flinders University (www.findingmyway.org.au), with Flinders University as the administering institution.

### Participants

#### Inclusion criteria

Patients are eligible to participate if they have been diagnosed with any cancer (e.g., breast, prostate, colorectal, testicular, lymphomas, haematologic or gynaecologic malignancies); their cancer is being treated with curative intent; they are currently receiving active cancer treatment (surgery, chemotherapy, radiotherapy), or, if they received surgery alone, have been diagnosed within the last six months; they are aged 18 years or over; and they have sufficient English language literacy to understand the information sheet and consent form and navigate the website, access to the internet (home or work), and an active email address and phone number.

#### Exclusion criteria

Patients are ineligible to participate if they have a diagnosis of advanced cancer.

#### Withdrawal criteria

Patients who opt to withdraw from the study are asked if they would consent to continue completing follow-up measures, and for any existing data to be included in analyses. If consent is not given, their data will be erased from the database along with any documentation related to their involvement.

#### Recruitment

**Figure 1:**
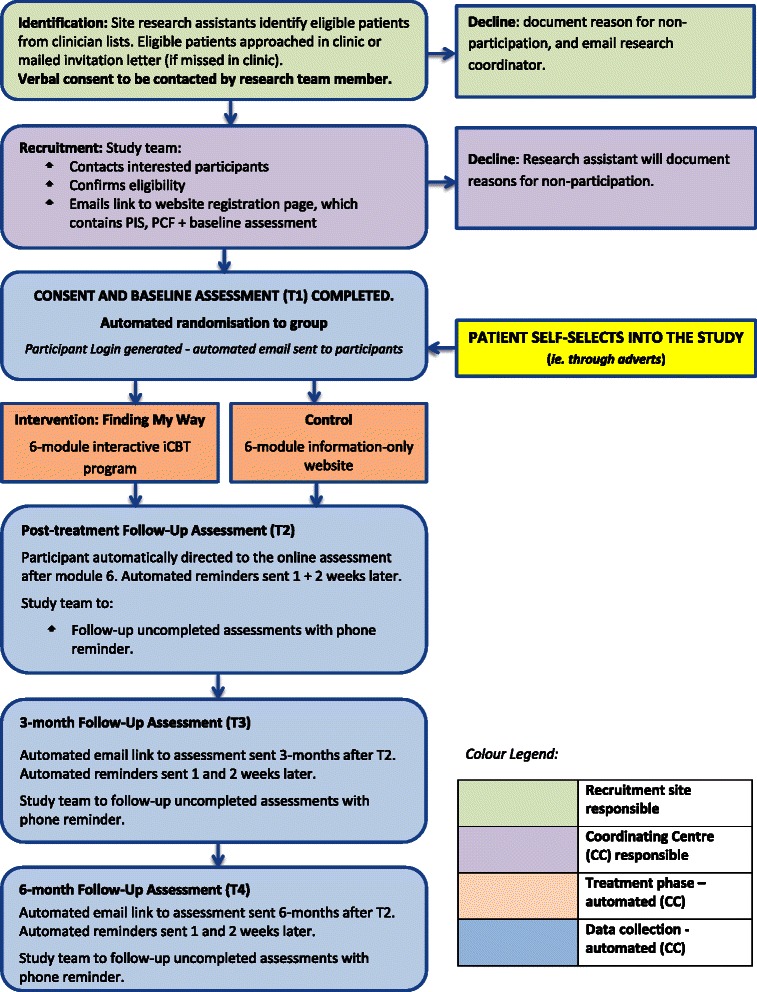
Flow chart of study.

An overview of recruitment and enrolment processes is provided in Figure 1. Participants are recruited from Australian hospitals and cancer centres according to any of the following methods:

1. **Self-referral:** Participants self-refer to the website in response to advertisements placed in local and national newspapers, on websites or in newsletters of cancer agencies or support networks, or in waiting areas of cancer agencies and clinics of participating hospitals.
2. **Direct approach:** Potential participants are approached directly by cancer clinicians or research assistants at participating hospitals, and are given a flyer containing a brief overview of the program and the website address. Verbal consent is obtained at this time for the research coordinator to provide either (i) a single reminder phone call, during which the research coordinator provides further information about the study and obtains the participants’ email address in order to send a direct link to the website, or (ii) when the patient cannot be contacted by phone, a single reminder letter providing further information about the study including the website address.
3. **Letters of invitation:** Letters printed on the site’s letterhead and signed by the department head are sent to patients missed in clinic. These letters ask patients to consider participation and allow contact by the researchers (via an opt-out response slip). A single reminder phone call is then made to all patients invited to take part in the study if they do not respond to the invitation letter after two weeks. Protocol at some sites requires that a clinician/ research assistant from the recruiting hospital contacts the patient to obtain verbal consent for patient details to be provided, prior to contact by the research coordinator.

#### Enrolment

Participants follow the email link or type in the web address in order to access the website and register for the program. Registration generates an automatic email to the participant containing a link to activate their account. Following account activation, participants are directed to read an online information page and consent form. Eligibility criteria are clearly highlighted on the information page, and consent requires participants to check a box stating they do not have advanced cancer. Consent is obtained by the participant clicking on an “I agree” button on the online consent form.

Following the consent process, participants complete the baseline questionnaire. Participants are then randomised to either the intervention or attention-control condition and are directed to a welcome tutorial and their user home screen, from which they are able to access their first module immediately. Participants work through 6 modules: one new module is accessible each week, along with previously accessed modules. The order of module release is self-determined during a goal-setting exercise conducted when first logging into the program, which allows participants to identify which topics are most immediately relevant to them, and can be subsequently modified if certain topics become more relevant as participants progress through the program. Participants receive automated email reminders to use the program each week. A booster module, comprised of a summary of the key information points from the program and links back to topics and worksheets, is accessible one month later. The opportunity to provide qualitative and quantitative feedback is provided at the end of each module.

#### Randomisation

This study is a parallel RCT, with equal numbers of participants randomised to the intervention and control arms. Randomisation is automated within the website; the random allocation sequence is generated by a researcher blinded to the identity of participants using computer-generated random numbers, and allocation is then automated by the website in a 1:1 ratio. Randomisation is stratified by gender.

#### Blinding

Participants are blinded to their intervention assignment; while they are aware there are two different versions of the website (explained as ‘information’ and ‘activities’ versions), they are not informed that one is a ‘control’ condition, are not aware which is the primary intervention being evaluated, and are blinded as to their own treatment group allocation. A list of blinded participant details is accessible to the research assistant for the purposes of conducting reminders about follow-up surveys.

### Intervention and control arms

#### Intervention

*Finding My Way* is a 6-week password-protected CBT internet application comprised of three main components: (1) psycho-education, (2) cognitive-behavioural worksheets/strategies including worksheets, quizzes, relaxation and meditation exercises, and (3) survivor testimonials. Each week a new module is released to participants, and participants can continue referring back to previous modules throughout the study.

The 6 modules address: (i) starting treatment – working with your medical team, covering assertive communication and decision making, (ii) coping with physical symptoms and side effects – including fatigue, pain, insomnia, and provides activity pacing worksheets, and relaxation audiotracks; (iii) coping with emotional distress – which covers depression, anxiety, anger and stress, and provides cognitive restructuring diaries, and mindfulness audio-tracks; (iv) body image, identity and sexuality – with psychosexual worksheets, and therapeutic writing activities; (v) your family and friends – comprising further assertive communication and needs assessment worksheets; and (vi) completing treatment, which includes self-management strategies to facilitate healthy lifestyles.

A private online note-taking feature, mood monitoring/management, and a resource section with links to reputable cancer-related organisations and other health websites are also available throughout the duration of the program. The program is outlined in Table 1.

**Table 1:** ***Finding My Way*** **content**

*Module*	*Topics covered*	*Worksheet/Activities*
Starting treatment	Working with your medical team, treatment planning	Assertive communication, Decision making
Physical symptoms	Coping with side effects including fatigue, pain, insomnia	Activity pacing, Relaxation exercises
Emotional distress	The emotional rollercoaster, including depression anxiety, anger and stress	Cognitive restructuring, Mindfulness exercises, Therapeutic writing
Identity	Concerns regarding body image, loss or changes in perceived identity, intimacy and sexuality	Psychosexual worksheets, Therapeutic writing
Family & Friends	The range of support concerns that arise, including identifying support people, impact on children and partners	Assertive communication, Needs assessment
Completing treatment	Commencing preventative health behaviours, follow-up care planning	Self-management, Goal setting
Booster	Summary of key themes from previous 6 topics. Links back to key webpages.	Links back to key worksheets (goal setting, cognitive restructuring, relaxation, communication).

#### Attention control

An information-only version of *Finding My Way,* developed to provide an appropriate attention control for the study, covers the same 6 module topics without any of the worksheets, activities, relaxation exercises, or note-taking feature. Our previous research indicates that an attention control condition does not significantly reduce distress [29], and provides a more rigorous test of the intervention.

### Outcomes

Primary, secondary and moderator outcomes are assessed using online self-report questionnaires. Baseline questionnaires are completed upon enrolment/ prior to randomisation. Post treatment questionnaires are completed at the conclusion of the sixth module (seven weeks after completing the baseline survey), and follow-up questionnaires are completed approximately 3 months and 6 months after treatment completion.

#### Primary outcome: distress

The primary outcome for this study is distress, measured as general distress and as cancer-specific distress. General distress is measured using the total scale score of the 21-item Depression Anxiety Stress Scale – short form (DASS: [33]), which assesses levels of anxiety, depression and stress over the previous week on a four-point scale ranging from (0) Did not apply to me at all to (3) Applied to me very much, or most of the time. Cancer-specific distress is measured using the 17-item Posttraumatic Stress Scale-Self Report [34], which measures on a 4-point scale (*0 = Not at all or only one time, 3 = 5 or more times per week / almost always*), the severity of each DSM-IV post-traumatic stress disorder symptom criterion in terms of how often respondents experienced each symptom in the previous week. For this study, the measure was adapted in order to reflect cancer diagnosis as the stressor.

#### Secondary outcomes

Secondary outcomes for this study include Health-Related Quality of Life (HRQOL), coping, and the economic impact of the intervention in terms of Health Service Utilisation. HRQOL is measured using the European Organisation for Research and Treatment of Cancer Quality of Life Core Questionnaire [35], a 30-item comprehensive health related quality of life assessment for cancer patients which yields a global QOL score, and five functional subscales (physical, emotional, social, role, cognitive).

Coping is measured with the mini-Mental Adjustment to Cancer Scale (mini-MAC: [36]), a 29-item scale yielding 5 factors: Fighting Spirit, Helplessness/Hopelessness, Anxious Preoccupation, Fatalism, and Cognitive Avoidance. Items are responded to on a 4-point scale ranging from ‘*definitely does not apply’* to ‘*definitely does apply*’.

Health Service Utilisation is examined using the 17-item Health Service Utilisation Questionnaire [37], which assesses health service use in the previous 12 months in terms of whether patients have been hospitalised and the number of visits to doctors and other health professionals. Hospitalisation is indicated as *no*/*yes day only*/*yes spent at least one night* (with number of days then recorded). Visits to GPs/hospital doctors/specialist doctors are indicated on a 7-point scale ranging from *none* to *25 or more times*, and utilisation of other health professionals (e.g. physiotherapist) is indicated as *yes* or *no*.

#### Moderators of outcomes

Participant and medical characteristics, availability of social support, information-seeking preferences, emotion regulation, vulnerability to cancer-distress, adherence to the program, and participant satisfaction are assessed as postulated moderators of program efficacy.

Participant and medical characteristics are assessed at baseline only, and consist of age, marital status, occupational status, annual gross income, level of educational attainment, area of residence (rural/urban, state), ethnicity, cancer type, time since diagnosis, treatment received (surgery, chemotherapy, radiotherapy, hormonal therapy, other), any other chronic health conditions, and other support-services accessed.

Availability of social support is assessed using the 20-item Medical Outcome Study Social Support Survey [38], which consists of four subscales: emotional/informational, tangible, affectionate, and positive social interactions, with availability of the support type listed in each item rated on a 5-point scale (*1 = none of the time, 5 = all of the time*).

Information seeking preferences in terms of the type and amount of cancer information sought by patients is measured by the Miller Behavioral Style Scale scale [39]. This scale identifies information seekers who look for and amplify threat-related cues (monitors) and distracters who avoid and minimize such cues (blunters), and has been used extensively in cancer populations. The scale asks the respondent to imagine two stress-evoking scenarios, each of which is followed by eight statements that describe different ways of coping with the stressor, with four statements being of a monitoring or information-seeking variety and four of a blunting or information-avoiding variety. Respondents are asked to check all statements that apply to them.

Vulnerability to distress is assessed using the Difficulties in Emotion Regulation Scale [40], a 36-item measure of difficulties in emotion regulation across six dimensions, namely a) lack of awareness of emotional responses, b) lack of clarity of emotional responses, c) non-acceptance of emotional responses, d) limited access to emotion regulation strategies perceived as effective, e) difficulties controlling impulses when experiencing negative emotions, and f) difficulties engaging in goal-directed behaviours when experiencing negative emotions. Participants respond to each item on a 5-point scale (*1 = almost never, 5 = almost always*).

Adherence to the study is measured in two ways. First, the self-help compliance scale [41] measures participant self-reported engagement with program’s (i) information, (ii) worksheets, and (iii) weekly time usage. Second, website use indicators are tracked on the website itself, including: number of visits, length of time logged in, and number of modules and worksheets completed.

Finally, levels of satisfaction with the website will be assessed as a measure of whether this intervention improved psychosocial support for patients. Qualitative feedback will be gathered within the website, and at follow-up assessments.

### Analysis

#### Sample size

Longitudinal sample size calculation for repeated measures was conducted using a program developed by Hedeker [42]. Previous online interventions targeting distress in community samples have effect sizes ranging from .42 to .65 for depression, and from .29 to 1.74 for anxiety [19]. Therefore, a conservative small-to-moderate effect size was selected for the current study. Based on the pilot RCT findings, an attrition rate of 21% at each of the assessment points can be expected [29]. Thus for a study with 2 groups and 4 assessment points, power set at 0.80, statistical significance set at α = .05 (two tailed), total attrition set at 21%, an effect size set at 0.35 SD, and an expected primary endpoint standard deviation of 4.0 at each time point [43], 94 participants per group are required, summing to a total sample of 188 participants.

#### Statistical analysis

Quantitative data will be analysed using the Statistical Package for the Social Sciences (SPSS) Version 17.0. Baseline differences between groups will be investigated using t-tests for continuous measures and *χ*^2^ tests of independence for categorical measures. Linear Mixed Effects Models (LMEM) will be employed to examine the efficacy of the intervention. The LMEM calculates a regression line for each individual while controlling for baseline observation (fixed main effects). Baseline observations will be entered as covariates to eliminate the influence of baseline variability, resulting in a 2 (group: intervention; control) X 3 (time: post-program; 3-month follow-up; 6 month follow-up) fixed effects model for each outcome variable, with random effects accounting for individual variation. This approach effectively equalises conditions at baseline and consequently allows for direct comparison between conditions at each follow-up point. In this context, (a) interactions between condition and time, (b) main effects of group and (c) post-hoc pairwise comparisons at each follow-up point are all indicators of intervention effects. This analysis offers the benefit of estimation maximisation; it provides joint modelling for each participant of observed and missing data based on maximising likelihood for population parameters as a function of the observed data. LMEM analyses are therefore robust with respect to handling missing data and unbalanced designs in longitudinal research as all participants with at least one observed *post-treatment* data point are included. That is, as baseline data is entered as a covariate, participants who do not complete any of the post-program assessments are not retained. To overcome this limitation and ensure this trial adopts a fully intention-to-treat design, multiple imputation will be performed for participants with only baseline-data. Therefore all participants, regardless of missing data at one or more assessment points, will be included in analyses and accurate parameter estimates obtained. Within-group effect sizes and reliable change indices will be calculated to provide a measure of the magnitude and clinical significance of changes respectively. To test for moderation, separate LMEM analyses will be conducted for each combination of moderator and outcome variable, where the moderator and group are entered in model 1 to control for main effects. The product term is then entered in model 2, to represent the interaction between group and moderating variable, and will indicate the moderating effect.

## Discussion

Internet-based self-help modalities represent one means of overcoming existing barriers to cancer patients accessing supportive care and psychosocial services, including the lack of such services within the public health system, particularly for rural patients, and barriers to attendance even where such services are provided. However, previous studies examining the efficacy of web-based self-help modalities for cancer populations have suffered from a range of limitations including (1) lack of an established psychological model (such as CBT) as the basis for the program, (2) lack of a control condition (3) small sample size and consequent lack of power for analyses, (4) lack of follow-up analysis over time, (5) examining efficacy of programs for one cancer type (i.e. breast cancer) only, as opposed to heterogeneous cancer types, and/or (6) examining programs implemented after completion of cancer treatment, as opposed to soon after diagnosis when distress tends to be highest and psychological treatment most warranted.

To our knowledge, *Finding My Way* is unique in offering an iCBT intervention applicable to individuals with heterogeneous cancer types soon after diagnosis. The large scale of this national multi-site RCT will therefore enable examination of the efficacy of such a program in reducing patient distress, improving coping and health related quality of life, and reducing health service utilisation in individuals with a range of cancer diagnoses. Furthermore, this study offers the important contribution of evaluating potential moderators of change. This information can be of benefit clinically when determining who to refer for online-interventions (versus who may be more suitably treated via other modalities) as well as benefitting future research via the refinement of inclusion and exclusion criteria, improving trial stratification and increasing power [30]. Indeed, it has been suggested that results of treatment trials are often weak because moderators of treatment are not examined, thus they should be part and parcel of any treatment study [30]; however, few studies have specifically examined moderators of psycho-oncology interventions.

If found efficacious, *Finding My Way* will represent an important means of overcoming common barriers to face-to-face therapy in a cost-effective and accessible way, which may particularly assist those residing in rural/remote areas. Integration of such a program into the supportive care offered to cancer patients within the health system will therefore be a promising avenue for reducing patient distress after cancer diagnosis, and consequently for decreasing the cancer burden both on individuals and within the health system.

## Abbreviations

CBT: Cognitive behaviour therapy
DASS: Depression anxiety stress scales
ES: Effect size
FMW: Finding My Way
HRQoL: Health related quality of life
iCBT: Internet cognitive behaviour therapy
ISG: Internet support group
LMEM: Linear mixed effects models
mini-MAC: Mini Mental Adjustment to Cancer Scale
QOL: Quality of life
RCT: Randomised controlled trial
SET: Supportive expressive therapy
SPSS: Statistical package for the social sciences
